# Menstrual psychosis – From myth to reality: A case report of periodic psychotic symptoms associated with the menstrual cycle

**DOI:** 10.4102/sajpsychiatry.v27i0.1650

**Published:** 2021-06-29

**Authors:** Waddah M. Alalmaei Asiri

**Affiliations:** 1 Department of Medicine, Psychiatry Unit, College of Medicine, King Khalid University, Abha, Saudi Arabia

## Introduction

Menstrual psychosis is characterised by the acute onset of psychotic, mood or behavioural symptoms during certain stages of the menstrual cycle. By definition, these symptoms exhibit in a relapsing-and-remitting pattern in concordance with the menstrual cycle.^1^ Menstrual psychosis is not considered a ‘specific disease entity’ in the current standard classification of mental health disorders. However, the temporal relationship between the onset and remission of the symptoms of menstrual psychosis to specific stages of the menstrual cycles has prompted many experts to regard menstrual psychosis as a unique disorder.^2^ In his monograph ‘Psychosis Menstrualis’, Krafft-Ebing first described and classified menstrual psychosis in 1878.^3^ Many case reports have been published and enriched the scientific literature about this disorder.^4,5,6^

Notably, Brockington argued that menstrual psychosis is a ‘morbid phenomenon related to bipolar disorder’ because of the similarities of their clinical features and the absence of valid diagnostic criteria for menstrual psychosis.^1^ Meanwhile, Shah et al. reported that the diagnosis of patients with brief psychosis that occurs around the menstrual cycle and resolves spontaneously should be defined as a ‘psychotic disorder not otherwise specified’.^2^

Despite the scientific controversy, both researchers agreed that antipsychotic treatment is usually ineffective, and that steroid hormones and clomiphene are better treatment options.^1,2^

## Case report

A 23-year-old unmarried Omani woman was brought to the psychiatric clinic by her parents, with a 3 year history of behavioural and mood changes. Her mother had observed that these cyclical behavioural changes occurred during certain times of the patient’s menstrual cycles. Three days prior to menses, the patient became depressed, isolated herself and cried for no obvious reason. Shortly after her menstrual bleeding, she became excited and moved in back and forth pattern. She appeared awake and aware but emotionally detached from surrounding with incomprehensive speech. She became suspicious of her mother and had attacked her on several occasions. She would spend the night looking out of the window, believing that her persecutors will appear at any moment. She was observed to mutter to herself excessively and would make gestures as if protecting herself against invaders. Her condition progressed over time and on day 3 of her menstrual cycle, she would become mute, look perplexed and fearful and would kneel (in a Muslim prayer manner) for a long time.

Her symptoms would begin to improve on day 5 of the menstrual cycle and would almost always be completely resolved by day 8.

During interview, the patient was in the fifteenth day of her menstrual cycle, and could not recall most of her symptoms, as described by her parents. However, she reported having ‘strange feelings’ prior to her menstrual bleeding and ‘weird experiences’ following. She remembered hearing an old woman talking to her and seeing lines of blood on the walls of her room with intact orientation and awareness throughout her presentation as mentioned by the patient and her family.

Her symptoms would almost always start 3 days prior to menstrual bleeding and resolve completely by day 8 of the menstrual cycle. During the interval between these episodes, the patient was reported to be asymptomatic, interactive and socially active. Her first menstrual cycle had occurred at the age of 14 and experienced regular cycles.

She had no relevant medical history and had no family history of mental health disorders. She had never smoked tobacco, drank alcohol or used drugs.

A physical examination was unremarkable and her medical workup, including a hormonal study, brain computed tomography (CT) scan and electroencephalogram were unremarkable for any underlying medical disorders. She was prescribed olanzapine 5 mg once daily and was offered an appointment in the clinic during her next cycle. Her symptoms had slightly improved and had disappeared on day 5 of the menstrual cycle.

However, she was still reported to have episodes of screaming and mood lability on the first day of menstrual bleeding. The olanzapine prescription was increased to 10 mg once daily and the patient is still undergoing psychiatric follow-up.

## Discussion

This is the first case of menstrual psychosis reported in the Arabian Gulf region. The nature of the patient’s symptoms, which correlated with the menstrual cycle, is compatible with the definition of menstrual psychosis as described in the current literature.^1,2,7^ Spontaneous and complete resolution of the patient’s symptoms, in addition to the periodic recurrence in rhythm with the menstrual cycle, makes bipolar disorder an unlikely diagnosis.

The pathophysiology of menstrual psychosis is still unclear. Published findings, such as reduced dexamethasone suppression, disturbed circadian changes in cortisol levels and an alteration in the Thyroid Stimulating Hormone (TSH) response have led to the belief that the hypothalamic–pituitary–adrenal system is involved in menstrual psychosis. Notably, these abnormalities are only detected during the active phase of menstrual psychosis and abate between episodes.^8^

Excess estrogen, unaffected by progesterone, is another hypothesised aetiological factor of menstrual psychosis, which is based upon the association of menstrual psychosis with the anovulatory cycle.^8,9^

Despite antipsychotic treatment having been suggested to be ineffective in another study,^1^ the patient reported in this case showed a good initial response to olanzapine. Olanzapine was used as a symptomatic treatment for this patient because of the physician’s limited experience with menstrual psychosis and a lack of evidence-based guidelines. Shah et al. used a low dose of trifluoperazine as an initial treatment for an Indian woman diagnosed with menstrual psychosis with good response.^2^ Moreover, Gerada et al. used an antipsychotic to treat a 34-year-old woman with menstrual psychosis, with excellent response.^10^

Experts in menstrual psychosis have suggested that treatment with hormones or menstruation-suppressing agents are possibly more effective treatments. Estrogen, progesterone, androgen and thyroxine have been successfully used as treatment in many cases.^1^ Furthermore, danazole and clomiphene have been used with excellent results.^8,11^ No randomised controlled trial studies have been conducted in this field and most of the evidence has been gathered from case reports.

## Conclusion

The clinical description of the patient outlined in this case report highlights the unique features of menstrual psychosis as a distinct entity. However, the controversy in the diagnosis and treatment of this disorder is still ongoing. Future studies may provide more answers as to the nature and treatment of menstrual psychosis and bridge these differences between psychiatrists.
